# A Reverse-Genetics Mutational Analysis of the Barley *HvDWARF* Gene Results in Identification of a Series of Alleles and Mutants with Short Stature of Various Degree and Disturbance in BR Biosynthesis Allowing a New Insight into the Process

**DOI:** 10.3390/ijms17040600

**Published:** 2016-04-22

**Authors:** Damian Gruszka, Malgorzata Gorniak, Ewelina Glodowska, Ewa Wierus, Jana Oklestkova, Anna Janeczko, Miroslaw Maluszynski, Iwona Szarejko

**Affiliations:** 1 Department of Genetics, Faculty of Biology and Environment Protection, University of Silesia, Jagiellonska 28, 40-032 Katowice, Poland; malgorzata.a.gorniak@gmail.com (M.G.); ewelina.glod1@wp.pl (E.G.); e.wierus@gmail.com (E.W.); miroslaw.maluszynski@us.edu.pl (M.M.); iwona.szarejko@us.edu.pl (I.S.); 2 Laboratory of Growth Regulators, Centre of the Region Haná for Biotechnological and Agricultural Research, Palacký University, and Institute of Experimental Botany, Academy of Sciences of the Czech Republic, CZ–783 71 Olomouc, Czech Republic; jana.oklestkova@upol.cz; 3 Institute of Plant Physiology, Polish Academy of Sciences, Niezapominajek 21, 30-239 Krakow, Poland; anna.janeczko@poczta.fm

**Keywords:** barley, biosynthesis, brassinosteroids, mutant, reverse-genetics, semi-dwarf, splicing

## Abstract

Brassinosteroids (BRs) are plant steroid hormones, regulating a broad range of physiological processes. The largest amount of data related with BR biosynthesis has been gathered in *Arabidopsis thaliana*, however understanding of this process is far less elucidated in monocot crops. Up to now, only four barley genes implicated in BR biosynthesis have been identified. Two of them, *HvDWARF* and *HvBRD*, encode BR-6-oxidases catalyzing biosynthesis of castasterone, but their relation is not yet understood. In the present study, the identification of the *HvDWARF* genomic sequence, its mutational and functional analysis and characterization of new mutants are reported. Various types of mutations located in different positions within functional domains were identified and characterized. Analysis of their impact on phenotype of the mutants was performed. The identified homozygous mutants show reduced height of various degree and disrupted skotomorphogenesis. Mutational analysis of the *HvDWARF* gene with the “reverse genetics” approach allowed for its detailed functional analysis at the level of protein functional domains. The *HvDWARF* gene function and mutants’ phenotypes were also validated by measurement of endogenous BR concentration. These results allowed a new insight into the BR biosynthesis in barley.

## 1. Introduction

Brassinosteroids (BRs) are plant steroid hormones playing an important role in regulation of diverse physiological processes, vegetative growth, reproduction and response to environmental stresses (for review, see [1,2]). The richest sources of BRs are pollen grains and immature seeds, whereas mature organs contain much lower concentration of these hormones (for review, see [3,4]). BR metabolism has been studied to the greatest degree in *Arabidopsis thaliana*, resulting in the identification of genes participating in BR synthesis [5,6], signaling (for review, see [7,8]) and degradation [9,10].

In Arabidopsis, the biosynthesis of BRs comprises several steps leading from episterol to brassinolide and its major part is divided into two, interconnected parallel pathways: early and late C-6 oxidation [11,12]. However, BR biosynthesis is far less elucidated in monocot crops. In dicot species, Arabidopsis and tomato, genes encoding BR-6-oxidases, which contain the Cytochrome P450 domains (clan CYP85) and catalyze synthesis of two most biologically active forms of BRs (castasterone and brassinolide), are duplicated. However, brassinolide was not identified in monocotyledonous plant species, even in mutants with defects in BR signaling in rice [13,14] and barley [15]. In rice, *OsDWARF* encoding BR-6-oxidase (CYP85A) is a single-copy gene and it was demonstrated that the encoded enzyme does not catalyze the synthesis of brassinolide, and therefore in this species BR synthesis is terminated at castasterone [16]. Recently, in maize, three genes involved in BR biosynthesis were identified: *DWF4* and *DET2* involved in early steps of BR synthesis [17,18] and *Brd1* (*Brassinosteroid-deficient dwarf1*) encoding BR-6-oxidase [19]. It has been reported by Makarevitch and coworkers [19] that similar to rice, the maize genome contains one copy of the gene encoding BR-6-oxidase, contrary to the genomes of several dicot species—Arabidopsis [20], pea [21] and tomato [22].

In barley, four genes implicated in BR biosynthesis have been identified [23,24]. Function of the *HvDWARF* gene, encoding brassinosteroid C6-oxidase involved in BR biosynthesis, was validated in this research by measurement of endogenous BR concentration in allelic mutants of the gene. The function of the gene has been characterized through identification of the semi-dwarf barley mutants, carrying missense mutations within conserved fragments of the coding sequence. These alleles were identified in two mutant forms, 522DK and 527DK, derived from variety “Delisa”. However, these analyses were conducted based on “forward genetics” methodology, physiological screening of the isolated semi-dwarf mutants and the analysis of the *HvDWARF* coding sequence only, without quantification of endogenous BR biosynthesis intermediates [23].

The objective of this research was to identify and analyze functionally genomic sequence of *HvDWARF* with the TILLING (Targeting Induced Local Lesions IN Genomes) strategy [25]. This strategy has been developed as an alternative to insertional mutagenesis. It may be applied to any plant species, regardless of its genome size, ploidy level and way of propagation. TILLING combines the application of classical (chemical) mutagenesis, the availability of a targeted gene sequence and high-throughput screening methodology for the identification of mutations in the target gene sequence [26]. Analyses involving molecular markers (Amplified Fragment Length Polymorphism, AFLP and Cleaved Amplified Polymorphic Sequences, CAPS) carried out previously in our laboratory, which were aimed at determining the spectrum and frequency of mutations induced by the chemical mutagens (the same as used for development of the HorTILLUS platform, applied in this study), indicated that majority of the induced mutations is located in non-genic regions of the barley genome (mostly repetitive sequences of retrotransposons) with the small portion of the mutations located in coding sequences of genes. Mutations of various genes characterized so far using the HorTILLUS platform are most frequently missense or silent, with nonsense ones constituting only a small fraction.

The aim of the performed TILLING analysis was to identify a series of new alleles of the *HvDWARF* gene, with mutations located in various parts of the encoded CYP450 domain in order to determine their influence on plant phenotype and site-specific effect of mutations. In the presented paper, the molecular analysis and the characterization of the new alleles are described, along with the physiological analysis of the identified mutants. Nomenclature of the newly identified alleles (*brd1* (*brassinosteroid deficient1*)) conforms to the nomenclature suggested for barley mutants by Franckowiak and Lundqvist [27] and corresponds to the alleles (*brd1*) identified in the homologous genes from rice [28,29] and maize [19].

## 2. Results

### 2.1. Identification of Mutations and Their Molecular Characterization

The identified genomic sequence of *HvDWARF* was deposited in GenBank database (HQ619227). Unlike rice *OsDWARF* (OJ1519_A12.12) (GenBank: AC097276) and maize *ZmBrd1* (GRMZM2G103773 in www.maizegdb.org) homologs, which contain nine exons and eight introns, the barley *HvDWARF* gene is composed of eight exons and seven introns. Third exon of the barley gene seems to correspond to the combined exons third and fourth of the rice and maize homologs, which suggests that during the evolution from the common ancestor the third intron of barley *HvDWARF* gene was lost, since the length of the homologous proteins is very similar in rice, maize and barley (469 aa, 465 aa and 472 aa, respectively) with identity of the proteins reaching 80% and *Expect* value E = 0.0.

As the result of the TILLING approach, a series of alleles in the *HvDWARF* gene was identified. Upon the analysis of 701 bp-long fragment of the gene in more than 3000 M_2_ plants 2,153,472 nucleotides were screened. Thirteen mutations have been identified and this number includes missense, silent ones as well as the mutations located in the introns of the gene. No nonsense mutation was identified. The missense mutations and the ones located in the introns are listed in Table 1. 

**Figure 1:**
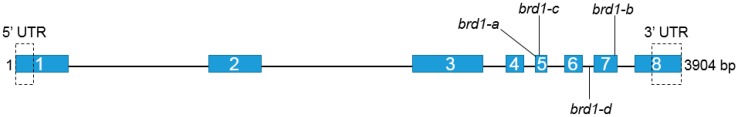
The *HvDWARF* gene structure with exons depicted as blue rectangles with consecutive numbers and introns as black line. Positions of the mutations identified in the *brd1-a*, *brd1-b*, *brd1-c* and *brd1-d* alleles are shown.

Out of the identified mutations, only two caused a BR-specific phenotype and height reduction of mutant plants, and therefore are described below in detail. Homozygous mutants carrying these two alleles were developed. The first of the newly identified alleles, *brd1-c*, carries a single-nucleotide missense mutation G3050T within the 5th exon (Figure 1). This mutation causes the change of arginine-347 to isoleucine. In this case, a basic, hydrophilic and positively charged amino acid residue (Arg) was substituted by a hydrophobic, non-polar one (Ile).

**Figure 2:**
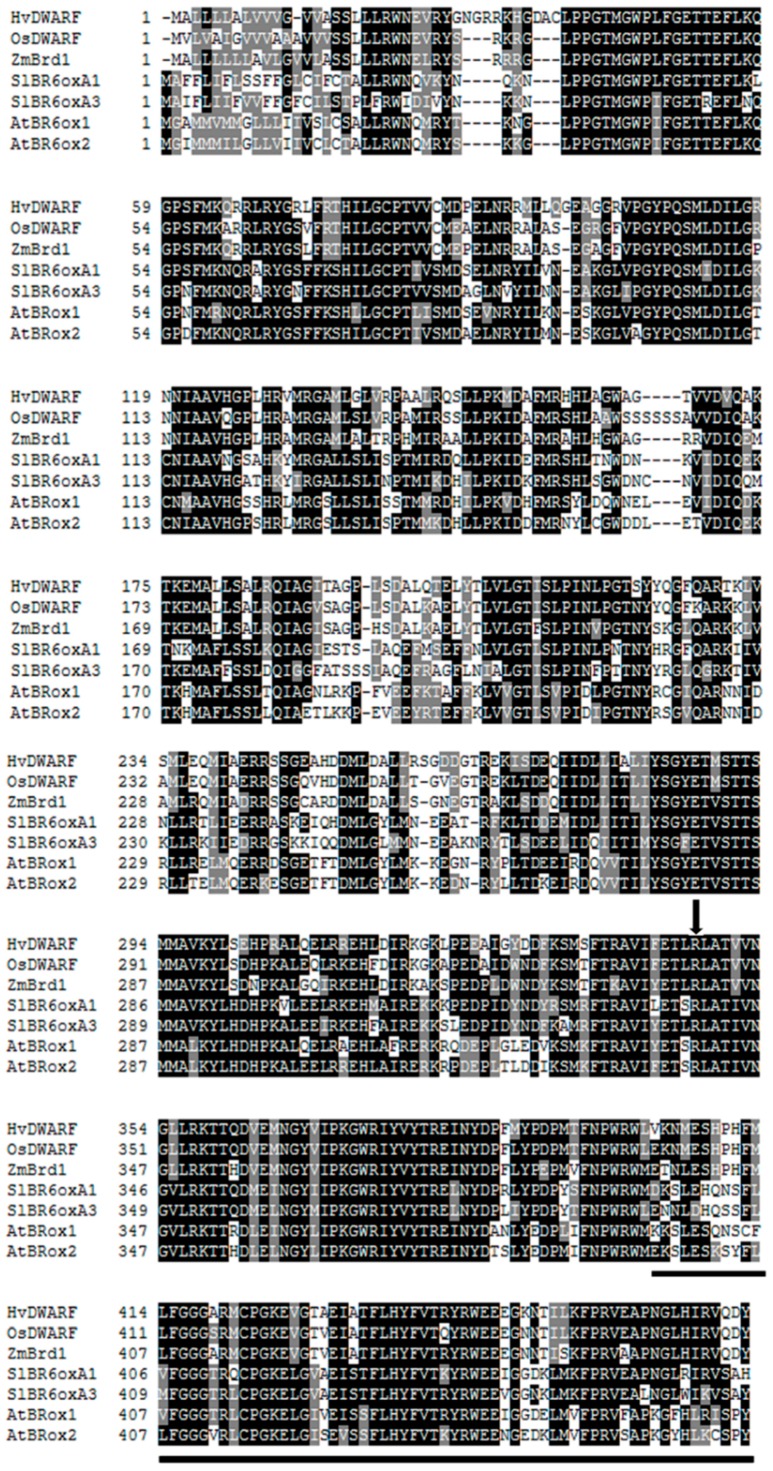
Alignment of the homologous proteins from *Hordeum vulgare* (HvDWARF, GenBank: AEG64639), *Oryza sativa* (OsDWARF, GenBank: AAT81671), *Zea mays* (ZmBrd1, GenBank: ACG46988), *Solanum lycopersicum* (SlBR6oxA1, GenBank: AAB17070 and SlBR6oxA3, GenBank: NP_001234520) and *Arabidopsis thaliana* (AtBR6ox1, GenBank: BAB60858 and AtBR6ox2, GenBank: NP_566852), performed with ClustalW and visualized in BOXSHADE. In case of *A. thaliana* and *S. lycopersicum* paralogous sequences were included. Highly conserved Arg-347, which is substituted by the mutation identified in the *brd1-c* allele, is indicated by the arrow, protein fragment whose sequence was altered as a result of the mutation in the *brd1-d* allele is underlined. In the barley HvDWARF protein the P450 cytochrome domain encompasses protein region 21–472. Black shade—fully conserved residue, grey shade—partially conserved residue.

The substituted amino-acid residue (Arg-347) is fully conserved at corresponding positions among homologous proteins from *Arabidopsis thaliana*, *Solanum lycopersicum* (tomato), *Oryza sativa* (rice), *Zea mays* (maize) and *Hordeum vulgare* (barley), as visualized in BOXSHADE (Figure 2).

**Figure 3:**
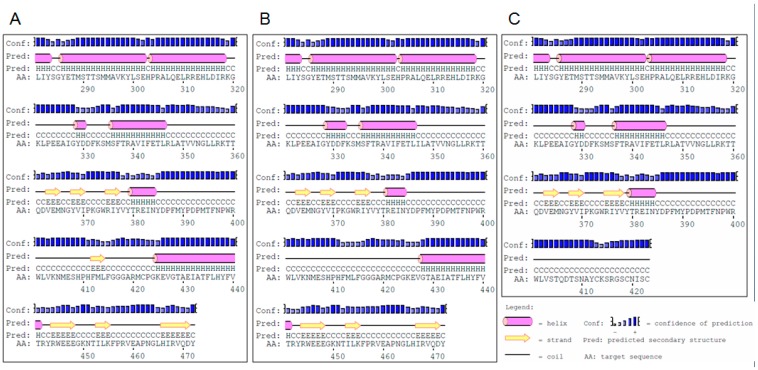
The arrangement of secondary spatial domains in the rearmost part of the HvDWARF proteins in the parent variety “Sebastian” (**A**); protein version encoded by the *brd1-c* allele (**B**); and in the truncated form encoded by the allele *brd1-d* (**C**).

The identified amino-acid substitution leads to rearrangement of the secondary spatial domains: loss of short β-sheet domain (present in wild type (WT) version of the protein at the positions 412–414) and shortening of α-helical domain by three positions in WT version of the protein, this domain occupies region 425–441, whereas in mutant version it spans the protein fragment 428–441 (Figure 3).

**Figure 4:**
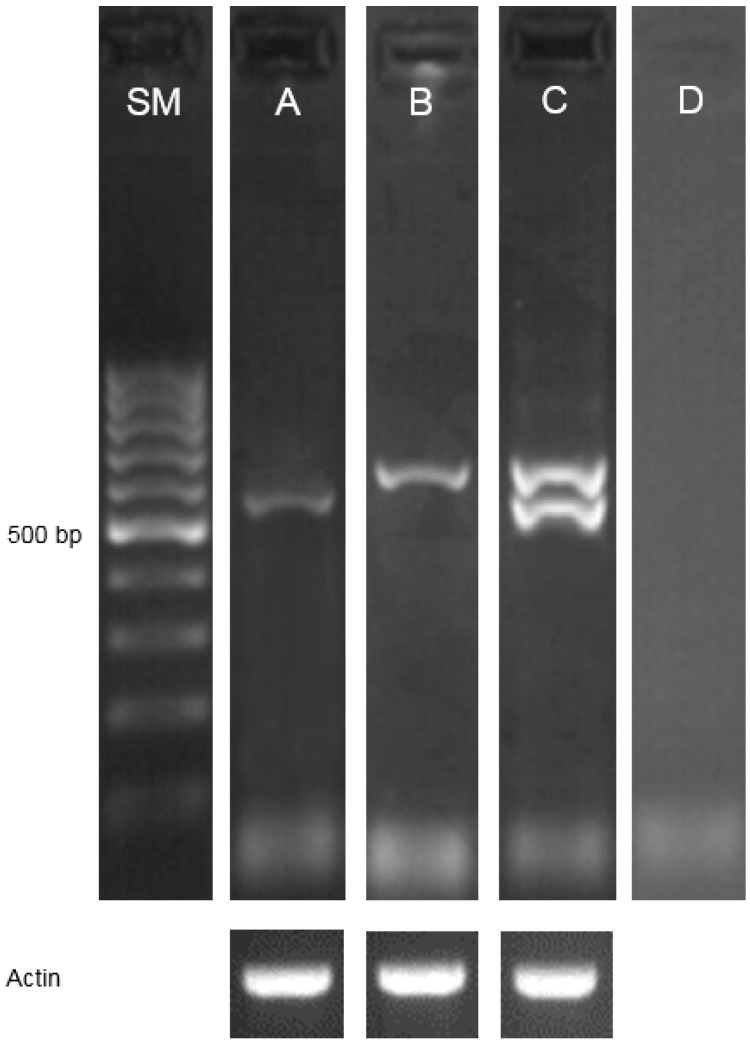
Reverse Transcription-Polymerase Chain Reaction (RT-PCR) amplicons produced on the basis of cDNA of the variety “Sebastian” (**A**); and the homozygous mutant *brd1-d* (**B**); RT-PCR reaction on cDNA of the heterozygote WT(Sebastian)/*brd1-d* producing double bands (**C**); the heterozygote shows normal phenotype. RT-PCR reaction without reverse transcriptase was used as a negative control (**D**). RT-PCR amplification of the barley actin mRNA (NCBI GenBank: AY145451) was performed as a positive control. SM—size marker, GeneRuler DNA Ladder Fermentas (100 bp), 1.5% agarose gel.

The second identified allele, *brd1-d*, carries the C3365T transition located in the 6th intron of the gene (Figure 1). The identified mutation is situated within the polypyrimidine tract of this intron. The influence of this mutation on the *HvDWARF* mRNA splicing process was determined. Reverse Transcription-Polymerase Chain Reaction (RT-PCR) reactions were performed on the basis of cDNA produced in reverse-transcription from mRNA extracted from the variety “Sebastian” and the mutant *brd1-d*. Reverse transcriptions were performed in three replicates for each genotype; in each case, RNA isolates were pre-treated with double volume (5 µL) (with respect to the original protocol) of (1 U/µL) RNase-free DNase in order to ensure complete elimination of any potential contamination with genomic DNA. Primers used in this experiment were designed to cover the transcript fragment containing exons surrounding the intron in which the mutation was identified. Upon agarose electrophoresis, the RT-PCR product amplified on the basis of cDNA of the “Sebastian” variety was ~570 bp long. However, the RT-PCR product amplified on cDNA of the homozygous mutant *brd1-d* was single-band and ~650 bp long (Figure 4). The single band of the RT-PCR product in the *brd1-d* mutant confirmed its homozygosity.

The RT-PCR products (from the variety “Sebastian” and the mutant *brd1-d*) were extracted from the agarose gel and sequenced. Sequencing results confirmed the difference in the RT-PCR products’ length, which is in accordance with the length (79 bp) of the intron 6, in which the mutation was identified. Therefore, it was concluded, that the mutation leads to the altered splicing of the transcript encoded by the *brd1-d* allele and retention of the 6th intron. The full-length cDNA of the *brd1-d* allele from the homozygous mutant and full-length cDNA from the “Sebastian” variety were cloned and sequenced. The sequencing analysis indicated that the identified mutation causing retention of the 79-bp-long intron within the *brd1-d* transcript was the only difference between the mutant and the WT “Sebastian” version of the transcript. Out of the mutations identified in various introns of the *HvDWARF* gene, the *brd1-d* allele was the only one resulting in splicing perturbation (Table 1).

Taking this into account, the *in silico* approach was undertaken to predict the sequence and secondary structure of the *brd1-d* mutant version of the HvDWARF protein. It was shown that the retained intron sequence causes a shift in open reading frame, introduces the premature stop codon, and consequently alters the C-terminal part of the protein sequence and its predicted secondary structure. The *brd1-d* mutation causes retention of the 79-bp-long intron sequence downstream of the position A-1210 in the *HvDWARF* coding sequence (total length of this CDS is 1419 bp). The predominant fragment of the P450 cytochrome domain (spanning protein region 21–472) remains unaltered in the mutant version of the HvDWARF protein (Figure 2), however downstream from V-403 a stretch of 20 amino acids <STQDTSNAYCKSRGSCNISC> is included, upon which translation is predicted to be terminated. The protein version encoded by this allele is therefore truncated by 49 amino acids. The identified mutation causes significant changes in the arrangement of secondary spatial domains in the rearmost part of the mutated protein. The mutant version of the protein is lacking a short β-sheet domain (positions 412–414), alpha-helical domain spanning positions 425–441 and three short β-sheet domains arranged consecutively at positions 444–448, 453–455 and 466–471 (Figure 3).

### 2.2. Phenotypes of the Mutant Plants and Physiological Analysis

The results obtained through molecular and computational analysis indicated that the identified mutations may have an impact on the phenotype of the mutant plants. Therefore, the homozygous mutant lines harboring the *brd1-c* and *brd1-d* alleles were isolated and phenotypes of these mutant lines were compared with the parent cultivar “Sebastian”. In order to confirm that the phenotypes of the identified mutants are caused by the identified mutations, each of the homozygous mutants was crossed with the parental variety, and the progeny was self-pollinated in order to analyzes the co-segregation of the mutants’ phenotypes with the mutations in F_2_ populations. Each of the alleles segregated in these populations as a monogenic, recessive mutation. 

For each of the identified homozygous mutants M_5_ generation has been developed, in each generation the homozygosity of the mutant plants was confirmed. The phenotypes of the identified homozygous mutants (of both alleles) were retained in the next generations of homozygous mutants (confirmed by the *HvDWARF* gene re-sequencing). No phenotype segregation was observed in the consecutive generations of both genotypes, indicating that their phenotypes are caused by the identified mutations. Allelism tests of the identified mutants and the mutants carrying the *brd1-a* and *brd1-b* alleles were also conducted. For each complementation (allelism) test 15 F_1_ plants were analysed. In each allelism test, the F_1_ plants showed mutant phenotype. Genetic analyses indicated that the newly identified homozygous mutants *brd1-c* and *brd1-d* are allelic to each other, and to the *brd1-a* (522DK) and *brd1-b* (527DK) mutants, validating that their phenotype is caused by mutation of the same gene—*HvDWARF*. Detailed characteristics of the identified mutants are given in Table 2.

**Figure 5:**
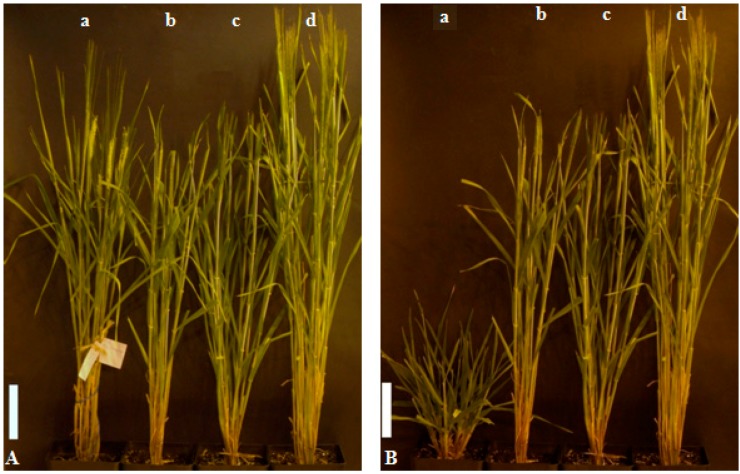
Phenotypes of the homozygous *brd1-a*, *brd1-b*, *brd1-c* and *brd1-d* mutant plants and the “Sebastian” variety: (**A**) *brd1-c* (**a**), *brd1-b* (**b**), *brd1-a* (**c**) and “Sebastian” (**d**); and (**B**) *brd1-d* (**a**), *brd1-b* (**b**), *brd1-a* (**c**) and “Sebastian” (**d**). Plants of all the genotypes are presented at the same developmental stage. Bar—10 cm.

Homozygous mutant with the *brd1-c* allele showed a relatively mild, semi-dwarf phenotype, the plant stature was similar to the mutants identified previously in the *HvDWARF* gene (alleles *brd1-a* and *brd1-b*) [23] (Figure 5). However, in the case of mutant plants with the *brd1-c* allele, a significant reduction in fertility was observed. The mutant plants produce spikes of the similar appearance as those of the parent variety, however only few grains per spike were fully developed. The reduction in fertility observed in the homozygous mutant *brd1-c* was correlated with its semi-dwarf phenotype and with the identified mutation, constituting an aspect of the phenotype of the homozygous mutant form. Microscopic analysis of anthers and pollen grains of this mutant with respect to the “Sebastian” variety indicated that the pollen grains of the *brd1-c* mutant are infertile. However, it should be emphasized, that heterozygous *brd1-c* mutants showed improved fertility; therefore, we postulate that it is an allele-specific effect.

The homozygous mutant with the *brd1-d* allele displayed a more severe phenotype: short stature (reaching only 30% of the height of the parent variety “Sebastian”), with proportionally shortened length and decreased width of the spikes (*ca.* 40% of the spike length of the parent variety) and proportionally reduced length of leaves (Figure 5). The mutant showed normal fertility. However, a delay in spike development was observed in the *brd1-d* mutant plants, which produced spikes 11–12 weeks after sowing, whereas the *brd1-c* mutant and the parent variety “Sebastian” produced spikes 9–10 weeks after sowing. The homozygous *brd1-d* mutant also shows a more intense color of tillers and leaves when compared with both *brd1-c* mutants and the parent variety plants. No difference in leaf morphology (curled or frizzled leaf blades) and width was observed between the identified mutants and the parent variety. There was no reduction in the number of tillers of the mutant plants of both genotypes when compared with the parent variety.

**Figure 6:**
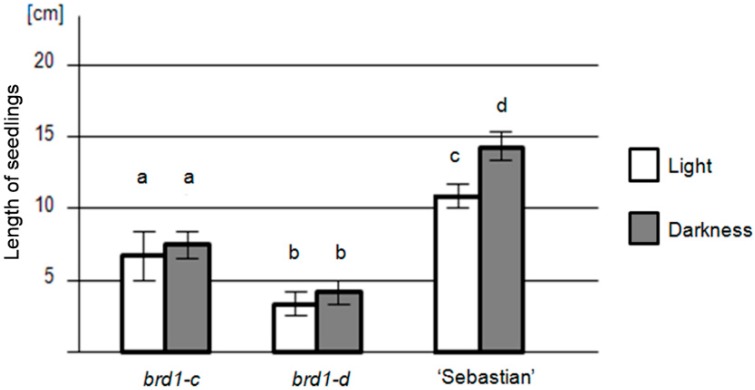
Results of the etiolation test for the identified mutants with the *brd1-c* and *brd1-d* alleles and the parent variety “Sebastian” (reference). Fifty seedlings per genotype were analyzed with three replications of the experiment. Values (seedling lengths) analyzed for each genotype were compared between the growth conditions (darkness *vs.* light). In the *brd1-c* and *brd1-d* mutants length of seedlings grown in the darkness is very similar to the length of those grown in the light (both mutants show lack of enhanced elongation during growth in the darkness), although the *brd1-c* seedlings were significantly longer than the *brd1-d* plants in the corresponding growth conditions. On the contrary, seedlings of the “Sebastian” variety grown in the darkness are significantly longer than seedlings of this genotype grown in the light. Error bars represent standard deviation. The differences between values denoted by the same letter are not statistically significant (*p* ≤ 0.05).

In order to characterize physiologically the identified mutants, the etiolation test and leaf-segment unrolling test were performed with the parent variety “Sebastian” as a reference. The identified mutants showed a lack of enhanced elongation of seedlings during the growth in darkness, and mutant seedlings grown in the darkness were of similar length to those grown in the light, which is a feature specific for BR mutants. The mutant seedlings of both genotypes grown in darkness and light were significantly shorter than the seedlings of the parent variety grown in corresponding conditions (Figure 6). This indicates that the growth retardation of the mutants begins early during the seedling development.

**Figure 7:**
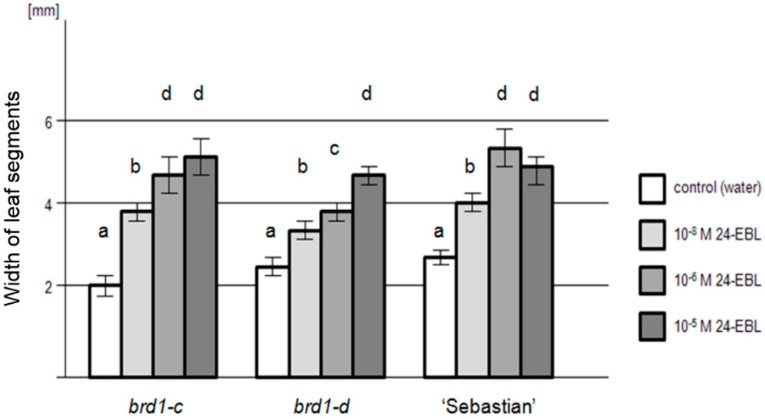
Results of the leaf-blade segment unrolling assay in response to various concentrations of 24-EBL for the identified mutants with the *brd1-c* and *brd1-d* alleles and the parent variety “Sebastian”. Ten leaf-blade segments per genotype per 24-EBL concentration were assayed in three replications of the test. Values obtained for a given incubation condition (control and various concentrations of 24-EBL) were compared between genotypes. Width of the leaf segments is given in millimetres. Error bars represent standard deviation. The differences between values denoted by the same letter are not statistically significant (*p* ≤ 0.05).

To confirm that the phenotypes of both mutants are attributed to the mutations identified in the *HvDWARF* gene (encoding the enzyme participating in BR biosynthesis) and the mutants are sensitive to the exogenous application of 24-Epi-brassinolide (24-EBL), the leaf segment unrolling test was performed. The leaf blades of the *brd1-c* and *brd1-d* mutants showed gradual unrolling correspondingly to the increase in the concentration of 24-EBL in the incubation solution, up to complete unrolling in response to the highest 24-EBL concentration. The lack of response in control treatment (water) was observed. Both mutants proved to be sensitive to exogenous application of BR. The leaf segments excised from mutant seedlings of both genotypes displayed a linear correlation between degree of unrolling and the hormone concentration, whereas leaf segments of the parent variety showed a decrease in unrolling at the highest concentration of 24-EBL (10^−5^ M), which may suggest a superfluous concentration of exogenous hormone (Figure 7).

In order to prove that the observed phenotype of the identified mutants is BR-specific, a phenotype-rescue assay was performed, in which the mutant plants of both genotypes (*brd1-c* and *brd1-d*) were treated with 24-EBL solutions of various concentrations (10^−8^ M, 10^−6^ M and 10^−5^ M) during the first three weeks of growth. Both mutants showed positive response to the treatment. Mutant plants with the *brd1-c* allele were of the same size as plants of the parent variety “Sebastian” (reference) when treated with both 10^−6^ and 10^−5^ M solutions of 24-EBL, however plants of the second mutant reached 90% of the parent variety size when treated with the highest concentration of 24-EBL after the three-weeks treatment.

The identified mutants were also treated separately with auxin (indole-3-acetic acid, IAA, 10^−5^ M) and gibberellic acid (GA_3_, 10^−6^ M) during early stage of seedling development as an additional test in the phenotype-rescue assay, in which the mutant plants of both genotypes (*brd1-c* and *brd1-d*) were treated with 24-EBL solutions of various concentrations during the first three weeks of growth under light condition. Auxin and gibberellic acid slightly increased the growth rate of plants, however, these hormones did not revert the mutant phenotypes. On the contrary, BR not only significantly stimulated growth of the mutants, but also reverted their phenotypes. At the end of this three-week experiment, plants of both mutant genotypes treated with high concentrations of 24-EBL resembled plants of the “Sebastian” variety (Figure S1).

**Figure 8:**
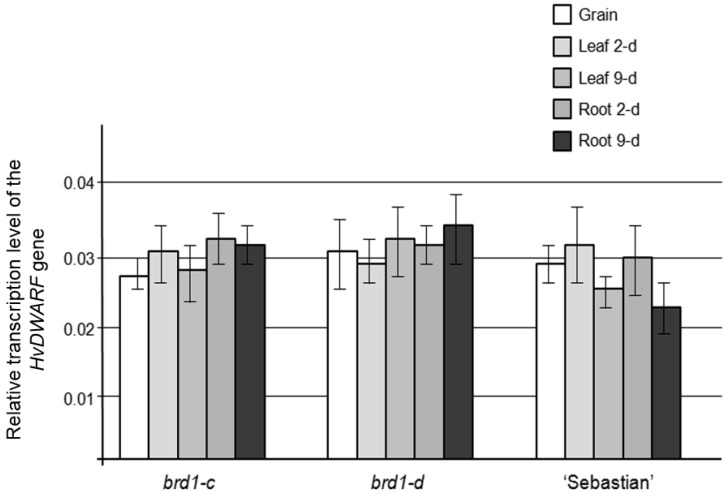
Transcription profile of the *HvDWARF* gene in the mutants with the *brd1-c* and *brd1-d* alleles and the parental variety “Sebastian” during early stages of barley seedling development. Transcription level of the reference gene *HvG3PD* (NCBI GenBank: X60343) on this diagram is equal to 1.0. Mean values of three replications are shown with error bars representing standard deviation. Values obtained for each of the developmental stages were compared between genotypes. Differences were not found statistically significant (*p* ≤ 0.05).

In order to analyze the transcription level of the *HvDWARF* gene in the identified mutants *brd1-c* and *brd1-d* and the parental variety “Sebastian”, the Real-Time Quantitative PCR technique has been applied. Spatial and temporal transcription pattern of the gene during early stages of seedling development was determined. The analyzed transcription level of the *HvDWARF* gene proved to be low and remained unaltered in the mutants with the *brd1-c* and *brd1-d* alleles, when compared with the parent variety. Transcription level of the gene was comparable (no statistically significant alterations were found) in all three genotypes irrespective of tissue analyzed or stage of seedling development (Figure 8).

### 2.3. Analysis of Endogenous BR Concentration

**Figure 9:**
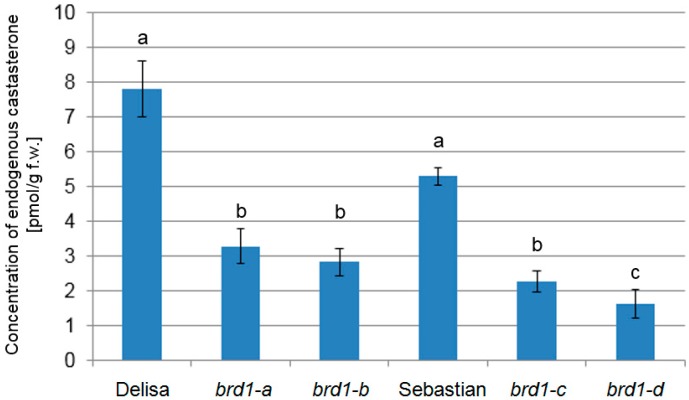
Concentration of endogenous castasterone measured in leaves of two-week-old seedlings. Mean values of three replications are shown with error bars representing standard deviation. Value obtained for each mutant was compared with its parental variety. The differences between values denoted by the same letter are not statistically significant (*p* ≤ 0.05).

In order to validate function of the *HvDWARF* gene in BR biosynthesis, content of endogenous castasterone (which is synthesized in reaction catalyzed by other BR-6-oxidases) was measured in the identified homozygous *brd1-c* and *brd1-d* mutants, and in the *brd1*-*a* and *brd1-b* mutants carrying missense mutations in the gene. The parent cultivar “Sebastian”, in which the *brd1-c* and *brd1-d* mutants were induced, and the parent cultivar “Delisa”, in which the *brd1*-*a* and *brd1-b* mutants were isolated, were used as reference genotypes in this assay. It should be emphasized that enzymatic role of the HvDWARF enzyme in castasterone biosynthesis has never been validated in this kind of assay. Results of this research confirmed the function of the HvDWARF enzyme and are congruent with the molecular, genetic and physiological data. The *brd1*-*a* and *brd1-b* mutants from variety “Delisa” contain similar, significantly lower content of castasterone in comparison with the parent cultivar. Similarly, the homozygous *brd1-c* and *brd1-d* mutants showed reduced concentration of endogenous castasterone in comparison with the parent cultivar “Sebastian”, however in the case of *brd1-d* (strong allele with predicted truncation of the encoded polypeptide) castasterone concentration was the lowest (Figure 9). These results are congruent with the effects of the identified mutations on the predicted protein sequence and structure.

## 3. Discussion

### 3.1. Effects of the Identified Mutations

The identified mutations result in different alterations of the predicted sequence of encoded polypeptide, are located in different parts of the cytochrome P450 domain, and therefore cause short stature of different degree. The *brd1-c* allele evokes a relatively mild, semi-dwarf phenotype of mutant plants. The phenotype of this mutant, in terms of plant architecture, is very similar to the previously identified semi-dwarf mutants *brd1-a* and *brd1-b* with single–nucleotide substitutions causing change of Val-341 to Ile and Gln-442 to Arg, respectively [23]. The amino-acid residues substituted in the *brd1-a* and *brd1-c* alleles are separated only by five residues. The substituted Arg-347 is located within <Glu-X-X-Arg> motif, which overlaps with Substrate-Recognition Site5 (SRS5) [20] what may reduce the enzymatic efficiency of the protein. Similarly to the presented mutant with the *brd1-c* allele, the alterations in the arrangement of secondary spatial domains were also reported in the semi-dwarf mutants *brd1-a* and *brd1-b* [23] what reflects the similarity in the mutants’ phenotypes. 

The mutation identified in the *brd1-d* allele is situated within the polypyrimidine tract of the 6th intron of the gene. The polypyrimidine tract in mRNA promotes assembly of the protein complex performing RNA splicing. The region is usually 15–20 bp long and located about 5–40 base pairs before the 3′ end of the intron to be spliced [30]. The identified mutation, influencing the splicing process, leads to the significant reduction of plant height.

However, similarly to what has been shown for the first two alleles of the *HvDWARF* gene, *brd1-a* and *brd1-b*, transcription level of the gene is very similar between mutants carrying the *brd1-c* and *brd1-d* alleles and the parental variety. Despite profound differences in the phenotypes of *brd1-c* and *brd1-d* mutants transcription level of the gene remains unaltered. It has been reported that the *HvDWARF* gene does not seem to compensate reduced level of BR biosynthesis, caused by mutations in its sequence, by elevated level of transcription [23] and it seems to be true also in case of the *brd1-c* and *brd1-d* alleles.

Development of multiple alleles of the gene with application of the “reverse genetics” strategy, like TILLING, allows detailed functional analysis of the gene and encoded protein by induction of point mutations and verification of an effect of amino acid substitutions in various domains of a protein on phenotype of a mutant. This insight is a valuable complementation and validation of information about conservation and role of individual amino acid residues, which could be inferred from alignment of homologous proteins and analysis of conservation performed with the use of bioinformatics prediction tools. Several amino acid substitutions identified in this study did not result in any changes of plant phenotype, even though the substituted amino acid residues were conserved among homologous proteins from various plant species. Taking into account a type of substitution, this kind of results constitutes an ultimate validation of a role of the individual amino acid residues. The homozygous *brd1-d* mutant identified in this study is, according to our knowledge, the first in which the dwarf phenotype and BR-deficiency are caused by abnormal splicing of the BR-biosynthesis gene transcript. Its physiological and biochemical analysis allowed a new insight into the process of BR biosynthesis in barley at the stage of castasterone biosynthesis.

Potentially, identification of new alleles of the genes involved in BR metabolism may be of interest for agronomic purposes. BR-deficient or insensitive mutants of various species (models and crops) show growth reduction of various extent. Especially in cereal crops, including barley, mutant-based breeding strategies, aimed at fine-tuning of the brassinosteroid biosynthesis and signaling pathways could improve plant phenotypes [2,24]. Moreover, different alleles of the same gene involved in BR metabolism may have various impacts on other aspect of plant physiology, even if the mutants present similar, semi-dwarf phenotype under optimal growth condition. This phenomenon was observed in the barley *uzu1.a* mutant, which carries a missense mutation in the *HvBRI1* gene encoding BR receptor. This mutant shows a heat-sensitive reduction in plant height, in contrast to allelic mutants harboring amino acid substitutions in other domains of the BR receptor [24]. Therefore, identification of new alleles of genes involved in BR metabolism in barley and other crops is of great importance both for basic and applied research. Identification of the series of alleles of the *HvDWARF* gene, with mutations located in various fragments of the gene, and resulting in plant growth reduction of various degree may serve as a tool of basic research. Indeed, the mutants presented in this paper will be a plant material of an experiment, which has just been commenced. Along with their parent cultivars the mutants will be exposed to drought, upon which various aspects of plant physiology and reaction to the stress condition will be analyzed, including efficiency of photosynthesis and CO_2_ assimilation, functioning of enzymatic and non-enzymatic antioxidant system and hormonal homeostasis. Participation of BRs in regulation of these processes has been suggested, therefore it may be assumed that the BR deficiency reported in the presented mutants should affect these processes, however differentially reduced size of the mutant plants may result in reduced water requirement, and therefore counterbalance this effect improving stress tolerance.

### 3.2. Comparison of the Mutations’ Effects and Phenotypes with Mutants of Homologous Genes in Other Monocots—Rice and Maize

Mutations with the profound effect on the homologous OsDWARF protein structure were identified also in rice [28,29]. The first allele, *brd1* (*brassinosteroid-dependent1*), contains 193-bp deletion in the *OsDWARF* gene, accompanied by short 5-bp insertion between exons 4th and 5th. As a result of this mutation, the shift in open reading frame occurs, leading to a disruption of the protein in the fragment from Asp-300 to Tyr-470, which renders the protein nonfunctional. The mutations caused severe dwarfism, extremely short leaf sheaths and short, curled and frizzled leaf blades. The mutant plants rarely produce short panicles with small, usually sterile seeds. Reduction in tiller numbers was also observed. The mutant also shows disturbances in skotomorphogenesis [29]. 

The other allele of the *OsDWARF* gene, *brd1-1*, has a 113-bp deletion from 5′ part of intron 6th to 5′ fragment of exon 7th. This deletion introduces a premature stop codon just downstream the deletion and causes formation of the truncated version of the protein. The mutant plants are severely dwarfed, reaching *ca.* 10% of the height of parent variety. The mutant develops the malformed erect leaves with stiff leaf blades and significantly shortened leaf sheaths, produces short panicles and shows abnormalities in skotomorphogenesis [28]. 

Mutant defective in the BR biosynthesis with mutation in the homologous *ZmBrd1* gene was characterized in maize. The single-nucleotide substitution introduces premature stop codon and profound truncation of the protein (mutant form is 165 aa long). The mutation causes a severe dwarf phenotype with marginal internode elongation, crinkled leaves and sterility of the *brd1-m1* mutant plants. The maize mutant form resembles the phenotype of the rice *brd1* mutants both in terms of plant height reduction and sterility [19].

Although several changes of phenotypic features are shared by the rice *brd1* and *brd1-1* mutants and the barley *brd1-d* mutant presented in this paper, it should be pointed out that in terms of both plant height reduction and grain production the barley mutant shows less severe phenotype (barley mutant does not show any reduction of fertility or leaf surface malformation). 

### 3.3. New Insight into the BR Biosynthesis in Barley

It is tempting to postulate that the difference in phenotypes between the severely dwarf rice and maize mutants, and reported in this paper barley mutant *brd1-d* results from the fact that rice and maize genomes contain only one copy of the gene encoding BR-6-oxidase [19,28,29], therefore loss-of-function mutations are not compensated by any other paralog. In this respect, barley genome is exceptional among monocots—it contains two genes encoding enzymes catalyzing C-6 oxidation reaction, leading to the biosynthesis of castasterone (CS) [23,24]. Both these genes are located in close vicinity in the telomeric region of the short arm of barley chromosome 2H. Therefore, identification of the *brd1-d* allele, which profoundly influences the structure of the HvDWARF protein, in this research shed more light on the BR biosynthesis in barley. In this mutant significant distortion of the predicted protein sequence and structure did not cause complete inhibition of castasterone biosynthesis—the content of endogenous castasterone was reduced by ~60% with respect to the “Sebastian” variety. However, in the severe rice mutants *brd1-1* and *brd1-2* castasterone was not detected [28], and the concentration of this compound was lowered more than ten times in another severe rice *brd1* mutant when compared with the wild type [29]. Less severe phenotype of the barley mutant *brd1-d* and relatively moderate (when compared with the rice *brd* mutants) reduction in castasterone biosynthesis indicate that the difference may result from partial compensation of the disturbance in CS biosynthesis by the paralogous gene—*HvBRD*. It was quite surprising to observe that barley mutants carrying nonsense mutations at various positions of the *HvBRD* gene (which also encodes the brassinosteroid-6-oxidase) retain relatively mild, semi-dwarf phenotype. In these mutants endogenous castasterone content was reduced by ~80% with respect to the reference variety [24], but this reduction is relatively moderate (when compared with the rice *brd* mutants). In this case perturbation in CS biosynthesis, caused by the nonsense mutations in the *HvBRD* gene, seems to be compensated by the other paralog—*HvDWARF*. All this indicates that in barley at the step of CS biosynthesis the two genes, *HvDWARF* and *HvBRD* may mutually, and, at least partially, compensate disturbances in their activity, which makes barley exceptional among other monocots—rice and maize—in which this step of BR biosynthesis is catalyzed by enzyme encoded by the single gene.

The reduction in concentration of endogenous castasterone was found statistically significant in the *brd1-d* mutant with respect to both the “Sebastian” variety and the *brd1-c* mutant (Figure 9). However, taking into account significant distortion of the *brd1-d* version of the protein, we postulate that in the *brd1-d* mutant castasterone biosynthesis may result from secondary, compensational castasterone biosynthesis by the paralogous enzyme—HvBRD. It is also possible that the reduced level of castasterone in the *brd1-d* mutant is too low to induce higher growth rate, and only exogenous application of 24-Epi-Brassinolide at high concentration (10^−5^ M) stimulates growth at the rate sufficient for reversion of the *brd1-d* mutant phenotype, whereas in the *brd1-c* mutant the corresponding phenotype reversion was induced by lower concentration (10^−6^ M) of 24-Epi-Brassinolide (Figure S1).

## 4. Experimental Section

### 4.1. The HorTILLUS Population

The barley (*Hordeum vulgare*) TILLING population (HorTILLUS) developed at the Department of Genetics, University of Silesia was employed during the identification of the new alleles of the *HvDWARF* gene. The population has been developed by a mutagenic treatment of grain of the barley variety “Sebastian” consecutively with two chemical mutagens: sodium azide (1.5 mM/3 h) and *N*-methyl-*N*-nitrosourea (MNU) (0.5 mM/3 h). A six-hour inter-incubation period between mutagenic treatments was applied. To establish M_2_ generation only one grain from each M_1_ plant was sown. For establishing the TILLING platform, DNA were extracted from leaves of more than 10,000 individual M_2_ plants and arrayed in eight-plant pools. 

### 4.2. Identification of the Genomic Sequence of the HvDWARF Gene and TILLING Analysis

The *HvDWARF* genomic sequence was identified based on mRNA of the gene (GenBank: DQ832258) and based on comparison with mRNA and genomic sequence of the homologous rice gene *OsDWARF* (GenBank: AC097276). Four primer pairs were designed using the Jellyfish software (LabVelocity, San Francisco, CA, USA) and applied for amplification of fragments of the *HvDWARF* genomic sequence. The primer sequences and PCR profiles are available in Supplementary materials (Table S1). The *HvDWARF* gene fragment assigned for the TILLING analysis encompassed gene part in the range 2843–3543 bp, comprising exons 4–7 and was flanked by short sequences of intron 3rd and 7th. It was delineated to cover the gene fragment encoding the most conserved region of the HvDWARF protein. Sequences of primers applied in the TILLING analysis and PCR profile are available in Supplementary materials (Table S2). The TILLING procedure was performed according to the protocol by Till and coworkers [31] with minor modifications. For cleavage of DNA, heteroduplexes 0.1× Celery Juice Extract (CJE) containing CEL I enzyme was used, kindly provided by B.J. Till (IAEA Laboratories, Seibersdorf, Austria).

### 4.3. Computational Analyses, Molecular Procedures and Physiological Tests

Position and length of exons, introns and UTR sequences within the identified *HvDWARF* genomic sequence were verified using the Spidey software [32] (National Center for Biotechnology Information: Bethesda, MD, USA). The ASD—Alternative Splicing tool (The European Bioinformatics Institute: Cambridge, UK) [33,34], which is now part of the ASTD—Alternative Splicing and Transcript Diversity Databases, was applied to determine the position of mutation (allele *brd1-d*) with respect to the donor site, branch point, polypyrimidine tract and acceptor site of the intron. The conservation of amino-acid residues was determined using ClustalW [35] (The European Bioinformatics Institute: Cambridge, UK) and visualized using the BOXSHADE program [36] (SIB Swiss Institute of Bioinformatics: Lausanne, Switzerland). The PSIPRED program [37] (University College London: London, UK) was applied to predict the influence of the identified mutations on secondary structure of the protein. 

RNA extraction was carried out using TriPure Isolation Reagent (Sigma-Aldrich, Poznan, Poland). Reverse transcription was performed with RevertAid Kit (Fermentas, Gdansk, Poland), with double volume (5 µL) of (1 U/µL) RNase-free DNase (with respect to the original protocol) for RNA isolate treatment. 

Analysis of *HvDWARF* transcription level was performed according to the procedure described previously [23] using the LightCycler^®^ 480 II analyzer (Roche, Warsaw, Poland). Relative transcription level of the *HvDWARF* gene in the identified mutants and the parental variety “Sebastian” (reference) was analyzed applying the LightCycler^®^ 480 SW software (version 1.5) (Roche). The analysis of the Real-Time QPCR data was conducted according to the method by Schmittgen and Livak [38]. 

Physiological tests were performed in three biological replications. Etiolation test was performed according to the protocol by Gruszka and coworkers [23]. In this test 50 seedlings per genotype/replication were analyzed both in the light and darkness. In the leaf segment unrolling test, performed according to the protocol by Chono and coworkers [15], 10 leaf segments per genotype/concentration/replication were measured. Results of the experiments were analyzed statistically applying the Student’s *t*-test and ANOVA. In the etiolation test values (seedling lengths) analyzed for each genotype were compared between the growth conditions (darkness *vs.* light). In the leaf-blade segment unrolling assay, values obtained for a given incubation condition (control and various concentrations of 24-EBL) were compared between genotypes. During analysis of the transcription profile of the *HvDWARF* gene values obtained for each of the developmental stages were compared between genotypes. In the analysis of the concentration of endogenous castasterone, value obtained for each mutant was compared with its parental variety. The mean values were subjected to the statistical analysis using the Student’s *t* test (*p* < 0.05).

### 4.4. Measurement of Endogenous Castasterone Concentration

Measurement of the endogenous BR biosynthesis intermediate—castasterone was performed according to a modified protocol described in [39,40]. All plants were grown for 14 days at 17 °C. For each genotype, measurements were performed in three replicates. The leaf material (1.2 g of fresh weight) was homogenized in 20 mL 80% (*v*/*v*) cold methanol. After centrifugation, the supernatant was enriched in deuterium-labeled brassinosteroids (internal standards) and passed through a Strata X column (Phenomenex, Torrance, CA, USA). The bound fraction was eluted with acetonitrile and evaporated to dryness. Brassinosteroids were determined by ultra-high performance liquid chromatography with tandem mass spectrometry.

### 4.5. Hordeum Vulgare Plants Were Used in this Study

The barley mutants *brd1-c* and *brd1-d*, carrying mutations in the *HvDWARF* gene, were identified within the TILLING population (HorTILLUS) developed at the Department of Genetics, University of Silesia, Katowice, Poland. All barley mutants used in this study were identified at the Department of Genetics, University of Silesia and are deposited in the germplasm collection of this Institution.

## 5. Conclusions

Application of the TILLING approach allowed the detailed functional analysis of the *HvDWARF* gene with insight into a role of individual amino acid residues through identification of the series of mutations in different fragments of the Cytochrome P450 domain. This approach also led to identification and characterization of the first barley mutant deficient in BR biosynthesis, whose phenotype is caused by perturbation in the splicing process. The various alterations of the HvDWARF protein sequences in these genotypes are reflected by differences in the severity of plant height reduction. Based on the performed genetic, molecular and physiological analyses and phenotype comparison, it is postulated that in barley at the step of CS biosynthesis the two genes, *HvDWARF* and * HvBRD* may mutually, and at least partially, compensate disturbances in their activity, which makes barley exceptional among other monocots.

## Figures and Tables

**Table 1:** The mutations identified within the *HvDWARF* gene with the TILLING approach and their phenotypic effect in homozygous mutant plants. Asterisks (*) indicate the alleles described in details in the text. The previously identified alleles *brd1-a* and *brd1-b* are also included.

Mutation	Mutation’s Position	Mutation’s Effect	Phenotype
C2956T	Exon 4th	A340V	Normal
G3031A *brd1-a*	Exon 5th	V341I	Semi-dwarf
G3050T *brd1-c* *	Exon 5th	R347I	Semi-dwarf
G3071A	Exon 5th	G354D	Normal
G3094A	Exon 5th	D362N	Normal
C3277T	Exon 6th	P393S	Normal
A2964G	Intron 4th	‒	Normal
G3131A	Intron 5th	‒	Normal
C3196T	Intron 5th	‒	Normal
G3353A	Intron 6th	‒	Normal
C3365T *brd1-d* *	Intron 6th	Alteration in splicing	Dwarf
G3388A	Intron 6th	‒	Normal
A3504G *brd1-b*	Exon 7th	Q442R	Semi-dwarf

**Table 2:** Phenotypic characteristics of the homozygous mutants with the *brd1-c* and *brd1-d* alleles and the parent variety “Sebastian”. The mean values and standard deviations (SD) are presented. Ten plants per genotype at mature stage of the development were analyzed.

Genotype		*brd1-c*	*brd1-d*	“Sebastian”
Seedling length (cm)	in light	7.2 ± 2.5 (SD)	4.1 ± 1.1 (SD)	10.7 ± 1.5 (SD)
	in darkness	7.5 ± 2.1 (SD)	4.5 ± 1.2 (SD)	14.2 ± 1.7 (SD)
Culm length at maturity (cm)		48.3 ± 5.4 (SD)	17.8 ± 3.9 (SD)	65.4 ± 3.6 (SD)
Leaf length (cm)		17.8 ± 3.5 (SD)	8.8 ± 2.2 (SD)	20.4 ± 3.3 (SD)
Length of internodes (cm)	1st	11.7 ± 2.3 (SD)	4.5 ± 1.0 (SD)	16.5 ± 2.2 (SD)
	2nd	9.8 ± 1.8 (SD)	3.9 ± 1.2 (SD)	13.9 ± 1.2 (SD)
	3rd	9.2 ± 2.2 (SD)	3.4 ± 1.1 (SD)	11.5 ± 2.1 (SD)
	4th	8.5 ± 1.5 (SD)	3.4 ± 0.8 (SD)	9.4 ± 2.0 (SD)
	5th	7.0 ± 1.4 (SD)	2.7 ± 0.9 (SD)	8.9 ± 1.5 (SD)
	6th	1.3 ± 0.5 (SD)	0.8 ± 0.4 (SD)	2.5 ± 0.9 (SD)
Spike length (cm)		8.5 ± 2.1 (SD)	5.5 ± 1.9 (SD)	10.5 ± 2.5 (SD)
Awn length (cm)		11.4 ± 2.5 (SD)	6.7 ± 1.9 (SD)	12.5 ± 2.4 (SD)

## Supplementary Materials

Supplementary materials can be found at http://www.mdpi.com/1422-0067/17/4/600/s1.
