# Supplementary material for: A Reverse-Genetics Mutational Analysis of the Barley HvDWARF Gene Results in Identification of a Series of Alleles and Mutants with Short Stature of Various Degree and Disturbance in BR Biosynthesis Allowing a New Insight into the Process
Source: Int J Mol Sci. 2016 Apr 22;17(4):600. doi: 10.3390/ijms17040600 (PMC4849053; doi:10.3390/ijms17040600)
Supplement: Supplementary file 1 [file ijms-17-00600-s001.pdf]

Supplementary Material: A Reverse-Genetics Mutational Analysis of the Barley *HvDWARF* Gene Results in Identification of a Series of Alleles and Mutants with Short Stature of Various Degree and Disturbance in BR Biosynthesis Allowing a New Insight into the Process

Damian Gruszka, Malgorzata Gorniak, Ewelina Glodowska, Ewa Wierus, Jana Oklestkova, Anna Janeczko, Miroslaw Maluszynski and Iwona Szarejko

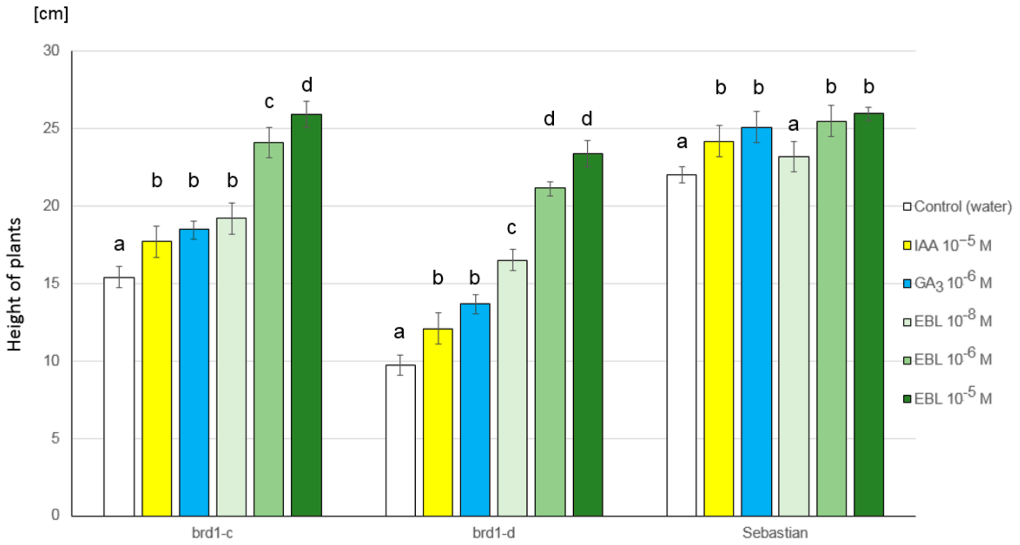

Figure S1. Results of the phenotype-rescue assay. The identified homozygous mutants *brd1-c* and *brd1-d* were treated in three-week experiment with various plant hormones, with Sebastian variety as a reference. Ten plants per genotype/treatment were analysed. Values obtained for each genotype after the treatment were compared with control value (water). Error bars represent standard deviation. The differences between values denoted by the same letter are not statistically significant ($p \leq 0.05$). IAA—indole-3-acetic acid, GA_3—gibberellic acid, EBL—24-Epi-Brassinolide.

Table S1. Primer pairs applied for amplification of fragments of the *HvDWARF* genomic sequence. T_a—temperature of primer annealing.

Primer Pair	Primer Sequence		T_a (°C)
	F	R	
DW1	5′GAGACCACCGAGTTCCTCAA3′	5′GATGTGCGTCCTGAACAGC3′	57.0
DW2	5′AAGCCTCCTCCCCAAGATG3′	5′AAGGGTGTACAGCTCTGTCTGC3′	57.5
DW3	5′AGATGGCATTGCTGTCTGC3′	5′TTTCCTCAGCAGACCATTGA3′	55.0
DW4	5′CGGCTACGACGATTTCAAGT3′	5′TCGTGTTCTTGCCTTCTTCC3′	54.5

PCR for primer pair DW1 was performed for 30 cycles (initial denaturation at 94 °C/3 min, followed by 30 cycles of 94 °C/45 s, 57.0 °C/45 s, and 72 °C/1 min 45 s for extension, with a final extension step of 72 °C/5 min).

PCR for primer pair DW2 was performed for 30 cycles (initial denaturation at 94 °C/3 min, followed by 30 cycles of 94 °C/45 s, 57.5 °C/45 s, and 72 °C/1 min 45 s for extension, with a final extension step of 72 °C/5 min).

PCR for primer pair DW3 was performed for 30 cycles (initial denaturation at 94 °C/3 min, followed by 30 cycles of 94 °C/45 s, 55.0 °C/45 s, and 72 °C/1 min 45 s for extension, with a final extension step of 72°C/5 min).

PCR for primer pair DW4 was performed for 30 cycles (initial denaturation at 94 °C/3 min, followed by 30 cycles of 94 °C/45 s, 54.5 °C /45 s, and 72 °C/1 min 45 s for extension, with a final extension step of 72 °C/5 min).

Table S2. Primers applied in the TILLING analysis and PCR profile. T_a-temperature of primer annealing.

Primer Pair	Primer Sequence		T_a (°C)
	F	R	
DW T	5′CTGATGATGGTTTCACCTTTGA3′	5′CAGGCGTGGATAAACAAAAGA3′	58.5

PCR for primers applied in the TILLING procedure was performed for 30 cycles (initial denaturation at 94 °C/3 min, followed by 30 cycles of 94 °C/45 s, 58.5 °C/45 s, and 72 °C/1 min 45 s for extension, with a final extension step of 72 °C/5 min).
